# Prognosis of nodal micrometastasis in resectable pN0 non-small cell lung cancer

**DOI:** 10.3389/fonc.2025.1424682

**Published:** 2025-01-31

**Authors:** Sophon Siwachat, Apichat Tantraworasin, Nirush Lertprasertsuke, Somcharoen Saeteng

**Affiliations:** ^1^ General Thoracic Surgery Unit, Department of Surgery, Faculty of Medicine, Chiang Mai University, Chiang Mai, Thailand; ^2^ Clinical Surgical Research Center, Chiang Mai University, Chiang Mai, Thailand; ^3^ Clinical Epidemiology and Clinical Statistic Center, Faculty of Medicine, Chiang Mai University, Chiang Mai, Thailand; ^4^ Department of Pathology, Faculty of Medicine, Chiang Mai University, Chiang Mai, Thailand

**Keywords:** resectable non-small cell lung cancer, nodal micrometastasis, occult micrometastasis, immunohistochemistry, prognosis

## Abstract

**Background:**

Nodal micrometastasis (NMM) is the presence of a small cluster of tumor cells in a regional lymph node. However, the prognostic value of NMM in resectable NSCLC is still debated.

**Methods:**

This retrospective cohort study at Maharaj Nakorn Chiang Mai Hospital from 2006 to 2017 assesses the prognostic impact of nodal micrometastasis in resectable pN0 NSCLC patients, using immunohistochemistry staining for cytokeratin AE1/AE3, p53, and BerEp4. Patients are categorized into three groups: pN0 without nodal micrometastasis, pN0 with nodal micrometastasis, and pN+. Overall survival is the primary endpoint, with disease-free survival as the secondary endpoint.

**Results:**

Out of 225 patients, 98 had pathological N0 (pN0) status and 127 had pathological N positive (pN+) status. Among pN0 patients, nodal micrometastasis was found in 21 cases (21.43%), distributed as follows: 2 (2.04%) in hilar and interlobar regions (N1), 17 (17.35%) in the mediastinal region (N2), and 2 in both hilar and mediastinal regions (N1+N2) (2.04%).Univariable analysis revealed that male sex and the presence of tumor necrosis increased both the recurrence rate of lung cancer and the mortality rate, whereas larger tumor size, intra-tumoral vascular invasion, and pleural invasion were associated solely with cancer recurrence. However, multivariable analysis showed no statistically significant difference in disease-free survival and overall survival between pN0 patients with and without NMM, with hazard ratios of 0.98 (95% CI: 0.31-3.08, P=0.973) and 1.11 (95% CI: 0.23-5.42, P=0.900), respectively.

**Conclusion:**

Nodal micrometastasis was identified in 21.43% of pN0 resectable NSCLC patients. However, the benefits of NMM detection in resectable cases remain controversial due to conflicting results from retrospective studies. Larger prospective cohort studies are needed to better understand disease prognosis and inform treatment strategies.

## Introduction

Lung cancer is characterized by its aggressive nature and poor prognosis, making it the leading cause of cancer-related mortality worldwide. In 2018, it accounted for 154,050 deaths in North America (1) and was the most common cause of cancer-related death in Thailand, accounting for 14.1% of all types of cancers (2). The presence of metastasis to regional lymph nodes is a significant prognostic factor for tumor recurrence and patient survival. The 5-year survival rates for non-small cell lung cancer (NSCLC) patients with pN0, pN1, pN2, and pN3 lymph node involvement were reported as 56%, 38%, 22%, and 6%, respectively (3). Despite thorough pre-operative mediastinal staging and complete oncologic resection, tumor recurrence has occurred in 30% of stage I and more than 60% of stage IIb cases (4). Moreover, there were also several recurrences in patients for whom pathological pN0 was confirmed after surgery. This suggests that nodal micrometastasis (NMM) to regional lymph nodes was under-detected during conventional histologic examination techniques such as hematoxylin and eosin (H&E) staining.

The American Joint Cancer Committee (AJCC) defines micrometastasis as a cluster of metastatic tumor cells greater than 0.2 mm but less than 2.0 mm, based on the size of the metastatic tumor cells. Clusters smaller than 0.2 mm and single cells are referred to as isolated tumor cells (ITCs) (5). According to the study conducted by van Schaik, the research focused on patients with stage I/II colon cancer with Duke A/B who underwent surgical treatment. A total of 137 patients were included in the study. The findings revealed that patients with nodal micrometastasis had a 5-year overall survival rate of 62%, whereas patients without nodal micrometastasis had an overall survival rate of up to 79%. Additionally, the disease-free survival rates were found to be 51% and 72% in patients with and without nodal micrometastasis, respectively (5). In breast cancer, the presence of NMM in the axillary lymph node is associated with unfavorable disease- free survival and overall survival outcomes. Currently, NMM is included in the TNM staging of AJCC breast cancer 8th edition, and its presence affects treatment planning (6).

In lung cancer, various antibodies such as AE1/AE3, Ber-EP4, and p53 have been utilized to detect metastatic cells in lymph nodes (7–9). However, the use of IHC staining for nodal micrometastasis detection and its impact on prognosis remain controversial topics that require further investigation. Wu et al. conducted a study on 103 patients with pN0 NSCLC, utilizing AE1/AE3 IHC staining to detect micrometastasis in resected lymph nodes. The findings revealed that 20.4% of patients had lymph node micrometastasis detected by special IHC staining, and these patients had poorer 5-year survival rates compared to those without micrometastasis (61.9% vs. 86.3%) (10). Conversely, Marchevsky and colleagues utilized AE1/AE3 staining to detect micrometastasis and concluded that NSCLC patients with LN micrometastasis had similar survivals to those with pN0 (11). Aim of this study is to evaluate impact of nodal micrometastasis detection through IHC staining on recurrence and survival in pathological N0 resectable NSCLC.

## Material and method

This is a dia-prognostic research conducted with a cohort design and retrospective data collection. The study was conducted on 225 patients with non-small cell lung cancer who underwent surgical treatment at Maharaj Nakorn Chiang Mai Hospital from January 2006 to December 2017. Among these patients, 98 individuals with negative pathological lymph node involvement (pN0) were designated as the study group, while 127 patients with positive pathological lymph node involvement (pN+) were included in the control group. The ethical considerations of this study were reviewed and approved by the Ethics Committee of the Faculty of Medicine, Chiang Mai University (study code: SUR-2562-06766). Due to the retrospective nature of this research, patient consent for reviewing their medical records was not mandated by the Institutional Review Board. All surgically treated patients underwent pre-operative staging, which involved CT chest imaging with upper abdominal coverage, transthoracic/transbronchial biopsy for tissue diagnosis, and mediastinoscopy for evaluating mediastinal lymph nodes prior to surgery. Due to cost limitations and reimbursement constraints in Thailand, endobronchial ultrasound (EBUS) and PET/CT were performed selectively in a subset of patients. Routine pre- operative evaluations such as 12-lead ECG, pulmonary function tests, and stair climbing tests were also conducted.

A comprehensive review was conducted on the computerized database and medical records of patients who underwent curative intent resection for non-small cell lung cancer during the study period. All surgical procedures were exclusively performed by a single surgical team under general anesthesia, employing either single lung ventilation with double lumen tube intubation or a single lumen tube in conjunction with an endobronchial blocker. The choice of surgical procedure, whether it be wedge resection, segmentectomy, lobectomy, bilobectomy, or pneumonectomy, depended on various factors, including tumor size, location, tumor staging, performance status, and the results of pulmonary function tests for each patient. In all cases, systematic mediastinal lymph node dissection was performed, including hilar, subcarinal, superior, and inferior mediastinal zones. Patients who had received pre-operative radiation or other systemic treatments, patients with a history of other cancer types, patients with insufficient follow-up data, and patients lacking pathological slides of lymph nodes were excluded from the study.

Pathological assessment of lymph node slides from patients in the pN0 group was performed by an experienced pathologist (N.L.) to confirm the absence of lymph node metastasis. Subsequently, tissue preserved in formalin and paraffin-embedded was sectioned at a 4-micrometer thickness. The slides are then deparaffinized, the first section was stained with hematoxylin to color the nuclei blue, and counterstained with eosin to color the cytoplasm pink. H&E staining was used to examine the morphology of the cells of interest. The second section underwent immunostaining using cytokeratin clone AE1/AE3 (diluted at 1:200), an antibody against cytokeratin, a protein abundantly present in the cytoplasm of cells originating from the epithelial cell lineage, which stains the cytoplasm brown. The third section was subjected to immunostaining with epithelial antigen clone BerEp4 (diluted at 1:100), which binds to the Epithelial Cell Adhesion Molecule (EpCAM) located on the cell membrane of epithelial cells, staining the cell membrane and/or cytoplasm brown. The fourth section was stained using p53 clone D0-7 (diluted at 1:5000), an antibody that binds to the p53 protein accumulated in the nucleus of cells with mutations in the p53 tumor suppressor gene, a mutation observed in many cancers. This staining renders the nucleus brown. These procedures was carried out using a Ventana automated immunostainer (Ventana Medical Systems, Tucson, AZ, USA), following the manufacturer’s specified protocol.

**Figure 1:**
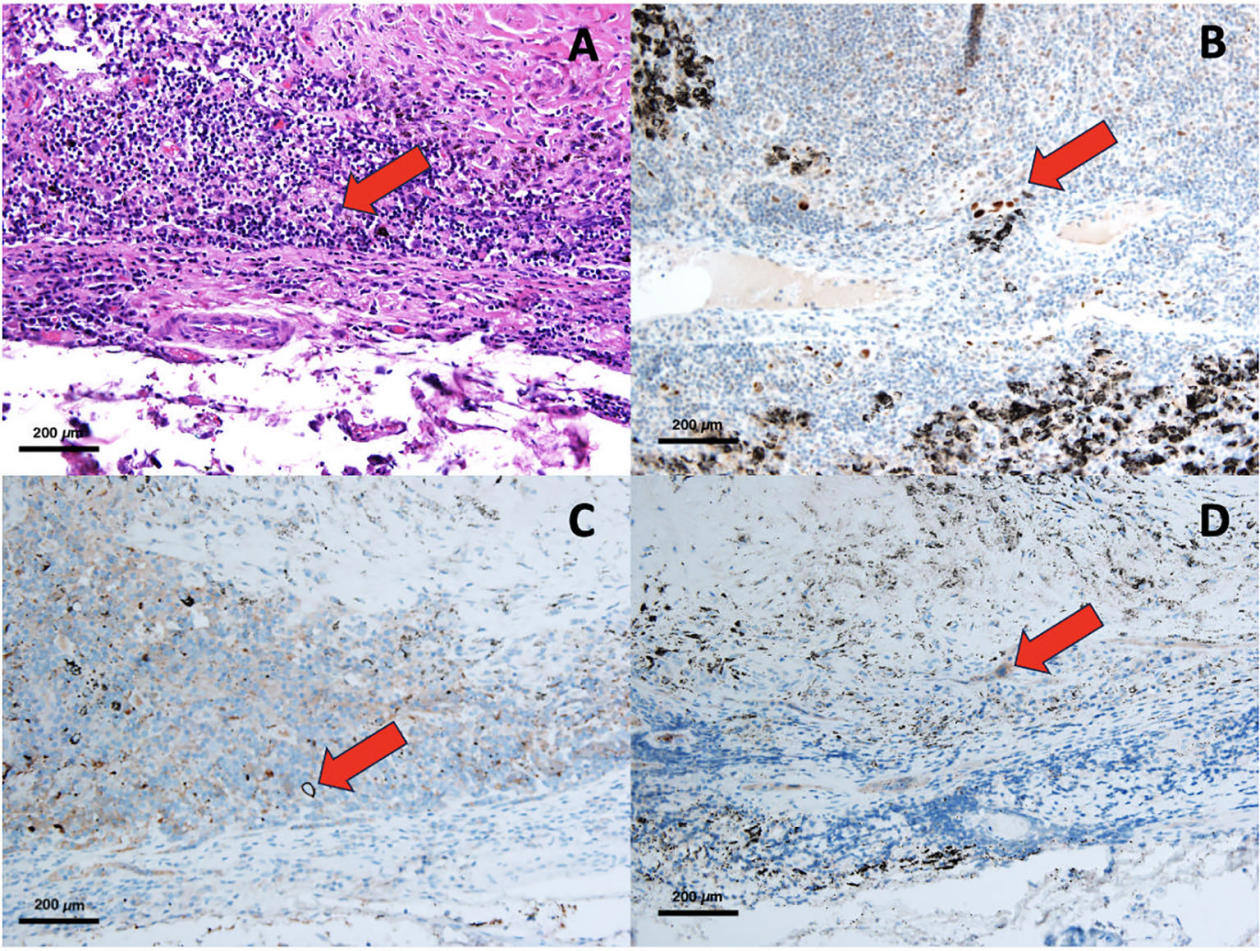
Pathological slide of NMM, H&E staining **(A)**, IHC with p53 **(B)**, IHC with Ber-Ep4 **(C)**, IHC with Cytokeratin AE1/AE3 **(D)**.

An experienced pathologist assessed the immunohistochemistry slides, completely unaware of the patients’ medical backgrounds and clinical statuses. The quantification of immunoreactivity was determined through the utilization of Fiji software, version 1.2. In this study, the diagnosis of nodal micrometastasis is based on the identification of at least one cell or a cluster of cells smaller than 2 mm that exhibit reactivity to at least one type of antibody. Additionally, in H&E staining, features consistent with cancer cells are observed, such as nuclear atypia, an increased nuclear-to-cytoplasmic ratio, prominent nucleoli, and hyperchromasia, among others. The primary endpoint of this study is the overall survival of patients, while the secondary endpoint is the disease-free survival of patients. Examples of nodal micrometastasis stained using various methods are presented in **Figure 1**.

Statistical analysis was conducted using STATA version 15.0. The sample size for this study was determined through power analysis, which was based on the findings of a prior study conducted by Jian Wu and colleagues (10). Continuous data were presented as either Mean and Standard Deviation (SD) or Median and Interquartile Range (IQR), depending on the distribution of the data. Categorical data were presented in terms of frequency and percentage. Continuous variables following a normal distribution were analyzed by t-tests, while non-normally distributed continuous variables were analyzed using the Wilcoxon rank-sum test. Multivariable survival analysis will be performed using the Cox proportional hazard model. Survival curves will be generated according to the Kaplan-Meier method, and statistical significance will be defined as a p-value less than 0.05.

## Result

**Figure 2:**
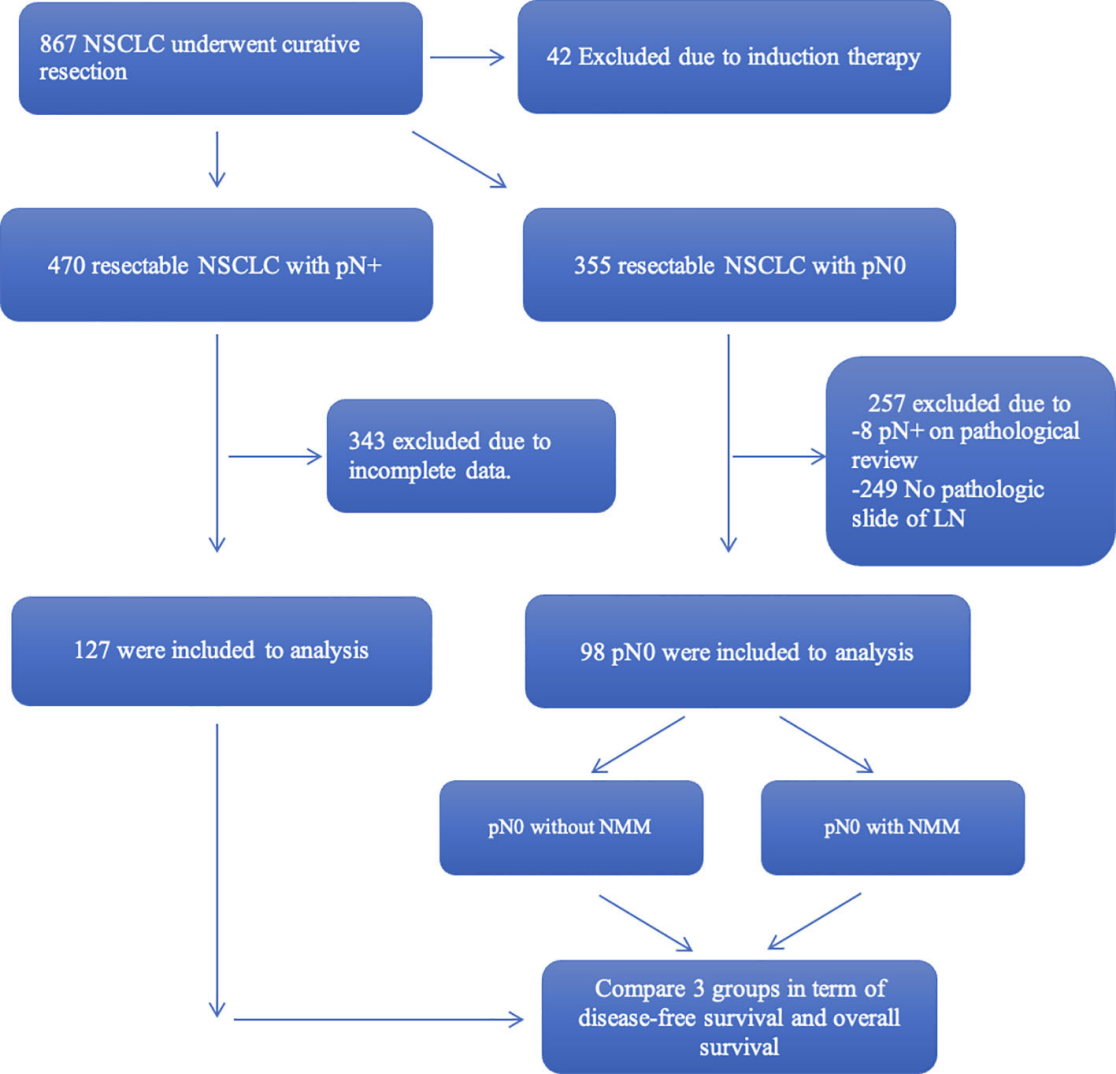
Study flow.

Over the study period from 2006 to 2017, a total of 867 patients diagnosed with non- small cell lung cancer (NSCLC) underwent curative surgical interventions at our institution. Among these patients, 42 individuals were excluded from the study due to receiving pre- operative systemic treatment before undergoing surgical resection, and 470 exhibited postoperative lymph node metastasis, as confirmed by histopathological examination. However, comprehensive data suitable for this study were only available for 127 cases, while the remaining 355 patients did not show evidence of lymph node metastasis. Out of the 355 pN0 patients, 249 individuals were also excluded due to the unavailability of pathological slides of lymph nodes for review. Moreover, an additional 8 patients were identified with lymph node metastasis during the review process. Thus, this study encompasses a total of 98 true pN0 patients included for analysis (**Figure 2**).

After performing immunohistochemical staining on the lymph nodes of 98 pN0 patients, nodal micrometastasis was found in 21 cases (21.43%). Among these cases, micrometastasis was exclusively confined to the N1 lymph node group in 2 patients, while 17 patients showed metastasis limited to the N2 lymph node group. Additionally, 2 patients were identified with nodal micrometastasis involving both the N1 and N2 lymph node groups. The details of individual patients with lymph node micrometastases are presented in **Supplementary Table S1**. The mean age of patients in the pN0 with nodal micrometastasis, pN0 without NMM, and pN+ groups was 62.81, 65.55, and 62.27 years, respectively. There were no statistically significant differences in terms of gender distribution, comorbidities, and surgical approaches among the patient groups. However, patients in the nodal micrometastasis group had significantly larger tumor sizes compared to the other two groups (p<0.001), with an average size of 6.13 centimeters (**Table 1**).

**Table 1:** Patient characteristic.

Characteristic	pN0 with LN micro-metastasis (21)	pN0 without LN micro-metastasis (77)	pN+ (127)	P value*
Age(y)(mean+/-SD)	62.81+/-9.26	65.55+/-10.12	62.27+/9.40	0.041
Gender (%)				
Male	12(60)	35(44.87)	77(60.63)	0.080
Female	8(40)	43(55.13)	50(39.37)	
Charlson index score	4.48+/-1.54	4.60+/-1.38	4.19+/-1.35	0.069
Tumor size Mean +/- SD	6.13+/-2.03	3.66+/-2.23	4.41+/-2.06	<0.001
Type of resection Sublobar resection Lobar resection	0(0) 21(100)	8(10.53) 68(89.47)	7(5.74) 115(94.26)	0.149

pN0, pathological lymph node negative; LN, lymph node; pN+, pathological lymph node positive; SD, standard deviation.

*p values were calculated using the Chi-squred test or ANOVA depend on type of parameter.

The majority of patients in this study had adenocarcinoma, comprising 74.67% of the cases. Comparing tumor characteristics, patients in the pN0 with NMM group demonstrated a tendency toward more poorly differentiated tumor histology, intratumoral vascular and lymphatic invasion, a higher degree of tumor necrosis, and a higher rate of receiving adjuvant systemic treatment compared to the pN0 without NMM group. The total number of lymph nodes retrieved during surgical procedures for the pN0 with NMM, pN0 without NMM, and pN+ groups was 21.85, 16.70, and 21.29 nodes, respectively (**Table 2**).

**Table 2:** Tumor characteristic.

Characteristic	pN0 with LN micro-metastasis (21)	pN0 without LN micro-metastasis (77)	pN+ (127)	P value*
Histology (%)				0.948
Adeno CA	17(80.95)	56(72.73)	95(74.80)	
Squamous cell CA	3(14.29)	18(23.37)	26(20.47)	
Other CA	1(4.76)	3(3.90)	6(4.73)	
Tumor differentiation				0.012
Well diff.	8(42.11)	41(59.42)	46(39.66)	
Moderately diff.	3(15.79)	22(31.88)	45(38.79)	
Poorly diff.	8(42.11)	6(8.70)	25(21.55)	
Vascular invasion	13(61.90)	21(27.27)	64(50.39)	0.003
Lymphatic invasion	20(95.24)	59(63.64)	120(94.49)	<0.001
Pleural invasion	6(28.57)	16(20.78)	28(22.05)	0.181
Perineural invasion	1(4.76)	4(5.19)	7(5.51)	0.338
Presence of necrosis	7(33.33)	17(22.06)	43(33.86)	<0.001
No.of LN retrieved (node/pt)(mean+/SD)	21.85+/-9.41	16.70+/-10.5	21.29+/12.16	0.009
Adjuvant treatment	15(71.43)	22(28.57)	95(74.80)	<0.001

pN0, pathological lymph node negative; LN, lymph node; pN+, pathological lymph node positive; SD, standard deviation; CA, carcinoma; diff, differentiation, pt, patient.

*p values were calculated using the Chi-squred test or ANOVA depend on type of parameter.

**Figure 3:**
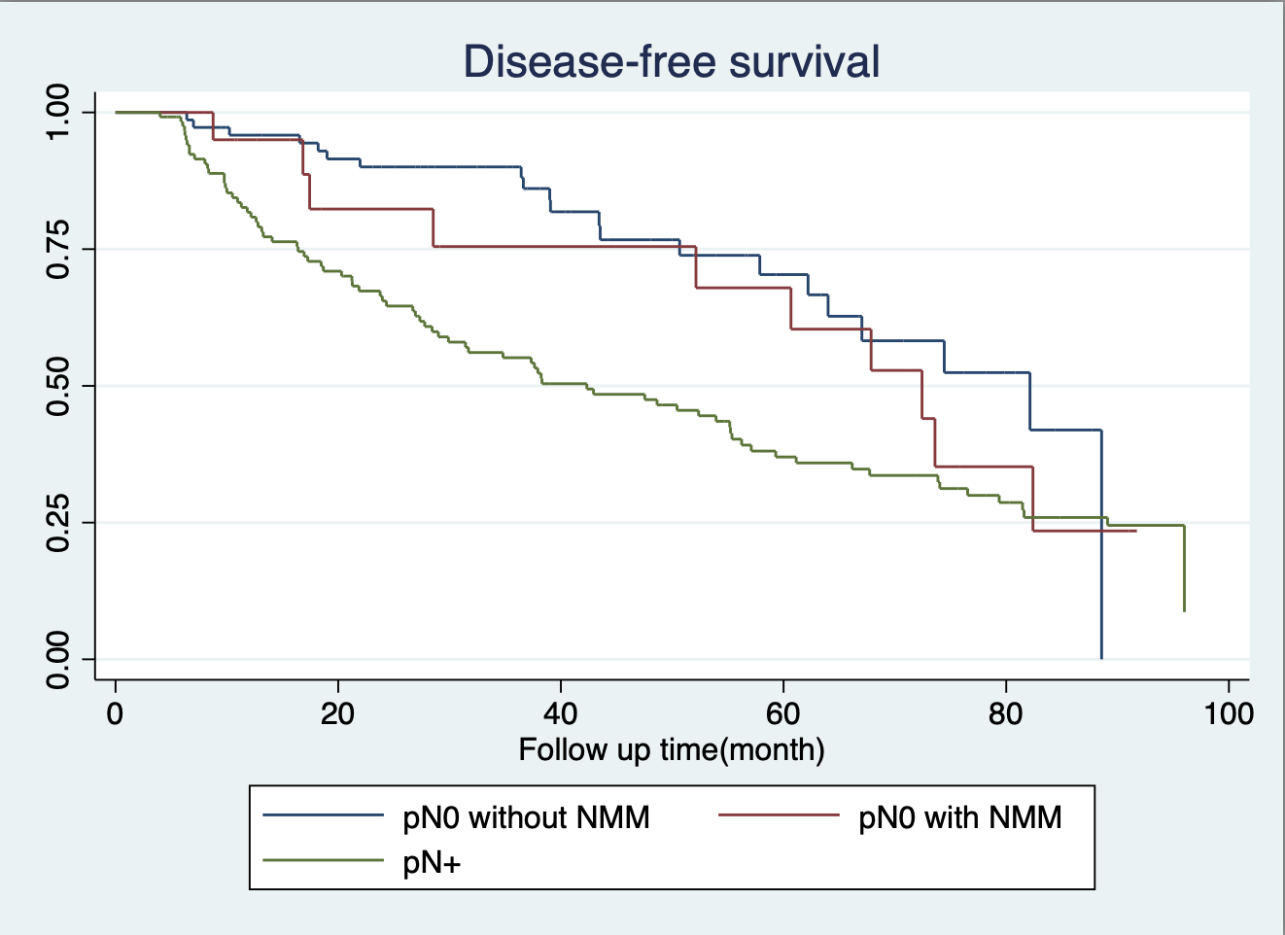
Disease- free survival.

**Figure 4:**
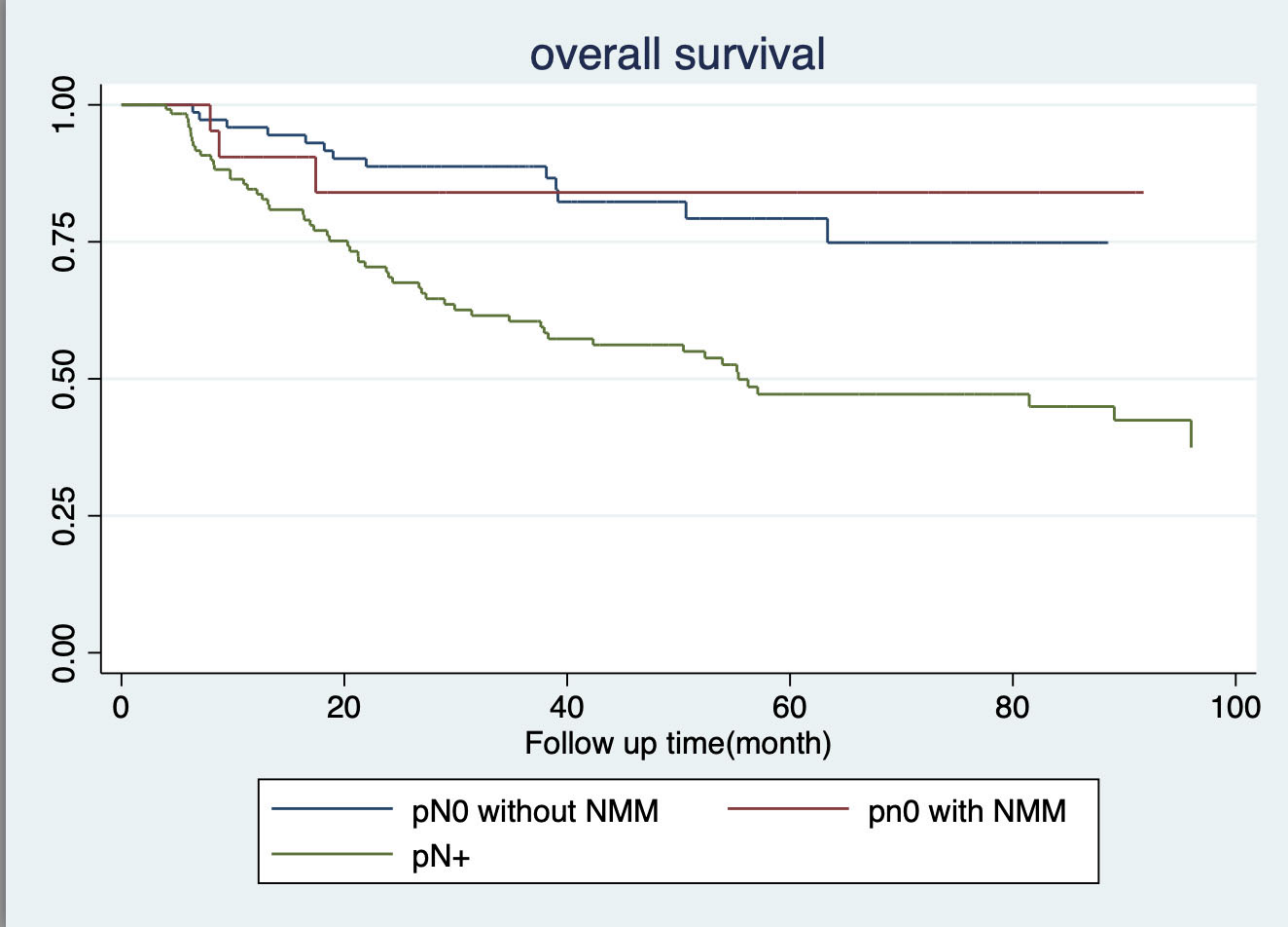
Overall survival.

In terms of 5-year survival, there were no statistically significant differences between patients in the pN0 subgroup, whether they had nodal micrometastasis or not, with survival rates of 84.01% and 70.26%, respectively. Similarly, regarding 5-year disease-free survival rates among pN0 patients with and without NMM, there were no statistically significant differences, both recording rates of 60.38% and 70.36%, respectively. Conversely, individuals in the pN+ subgroup exhibited notably lower rates of both 5-year survival and 5-year disease-free survival, at 47.19% and 37%, respectively, as depicted in **Figures 3**, **4**.

The results of the univariable Cox proportional hazard regression analysis for overall survival and disease-free survival revealed that male patients, the presence of larger tumors, and tumor necrosis were associated with poorer overall survival and disease-free survival. Meanwhile, intra-tumoral vascular invasion and pleural invasion were associated with poorer disease-free survival only. (Details are provided in **Supplementary Table S2**). The results of multivariable analysis are presented in **Table 3**. It was determined that the presence of nodal micrometastasis does not serve as a prognostic factor for tumor recurrence and lung cancer-related mortality among pN0 patients, with hazard ratios of 0.98 (95% CI: 0.31-3.08, P=0.973) and 1.11 (95% CI: 0.23-5.42, P=0.900), respectively. Conversely, patients in the pN+ group showed inferior 5-year survival and disease-free survival rates, with hazard ratios of 2.87 (95% CI: 1.06-7.77, P=0.038) and 4.77 (95% CI: 1.35-16.80, P=0.015), respectively. Details of the multivariable Cox survival analysis for other factors included in the model are presented in **Supplementary Table S3**.

**Table 3:** Multivariable analysis of disease-free survival and overall survival.

Characteristic	Adjusted hazard ratio	95% confidential interval	P value*
Tumor recurrence			
-pN0 without micro-metastasis -pN0 with micro-metastasis -pN+	1.00 0.98 2.87	Ref. 0.31-3.08 1.06-7.77	Ref. 0.973 0.038
Overall survival			
-pN0 without micro-metastasis -pN0 with micro-metastasis -pN+	1.00 1.11 4.77	Ref. 0.23-5.42 1.35-16.80	Ref. 0.900 0.015

pN0, pathological lymph node negative; pN+, pathological lymph node positive; Ref, reference.

*p values were calculated using the Cox-proportional hazard model.

In the subgroup analysis of patients with tumors smaller than 4 cm, it was found that the presence of nodal micro-metastasis in this patient group was associated with a higher recurrence rate compared to patients without micro-metastasis, with a hazard ratio of 8.28 (95% CI: 1.64-41.70, P=0.010). Conversely, there was no statistically significant difference in overall survival between pN0 NSCLC patients with and without micro-metastasis, with hazard ratios of 6.83 (95% CI: 0.76-61.74, P=0.087). As expected, patients in the pN+ group exhibited significantly worse overall survival and disease-free survival compared to pN0 NSCLC patients without micro-metastasis.

## Discussion

Lung cancer is generally associated with a poorer prognosis when compared to other cancers. The presence of metastasis in hilar and regional lymph nodes is regarded as a significant adverse prognostic factor. Survival rates of these patients progressively diminish when the disease spreads to lymph nodes farther from the primary site (3). Even among patients whom postoperative pathological examinations confirm the absence of cancer metastasis to the lymph nodes, resulting in recurrence rates as high as 30% in stage I patients and 60% in stage IIb patients (4). This finding implies that the detection of nodal micrometastasis in the regional lymph nodes was insufficient when using conventional histological examination techniques like hematoxylin and eosin staining. Despite the recognition of the substantial impact of cancer metastasis to the lymph nodes on disease prognosis, the established approach for identifying cancer within these nodes still predominantly relies on H&E staining a practice that has remained unchanged over an extended period.

The application of immunohistochemistry has proven to be a valuable tool in facilitate pathologists in the detection of subtle nodal metastasis which can be challenging to discern through H&E staining. This is largely attributable to the use of antibodies that target antigensfound on the surfaces of epithelial cells, typically absent in normal lymph nodes. Previous studies have revealed that the use of IHC staining can identify nodal micrometastasis in patients classified as pN0, with detection rates ranging from 9.3% to 44.9% (10, 12, 13). In this study, a panel of three different antibodies was utilized for immunohistochemistry staining with the aim of increasing the detection rate while minimizing the chances of false negatives.But, we detected a total of 21 cases of nodal micrometastasis out of the 98 pN0 patients. These findings closely match the previous study and did not significantly improve the detection rate. The antibody with the highest NMM detection rate is Anti-cytokeratin AE1/AE3, which can detect up to 25 out of 27 NMM positive nodes. Similar to the previous study, Anti-cytokeratin antibodies exhibit the highest detection rate (14–16).

Moreover, we also found that out of the patients with NMM there were 17 individuals (17.35%) exhibited cancer cell spread to the N2 group pf lymph nodes without involvement of N1, indicating the presence of microscopic skip metastasis. The analysis of data by Riquet and colleagues indicates that the incidence of macroscopic skip N2 metastasis, which refers to the presence of metastases larger than 2 mm in the N2 lymph nodes without any metastases in the N1 lymph nodes, ranges from 20.2% to 38%, with an average of 32.7% (17). There is currently a lack of consensus regarding the etiology of skip N2 metastasis, with potential explanations including aberrant pathways to mediastinal lymph nodes, unknown pathophysiological mechanisms, or the chance of metastasis being overlooked during routine pathological examinations. This raises questions about whether the true incidence of skip metastasis might be underestimated. In this study, it was observed that macroscopic skip N2 metastasis was identified in 41 instances out of 127 patients (32.28%) within the pN+ cohort. When including patients with microscopic skip N2 metastasis, the incidence of skip N2 metastasis increased to 58 cases, out of a total of 148 patients with nodal metastasis (39%) (both macro and microscopic metastasis. This incidence is higher than that reported in previous studies.

The presence of small amount of metastatic cell in lymph node may be eradicated by immune cells within the lymph node microenvironments. Hence, the detection of these metastatic cells may be only a transient phenomenon, or alternatively, these cells may evade he immune surveillance, survive and proliferation to form macrometastasis. Therefore, the prognosis of NSCLC patients with NMM is subject to considerable debate.

Studies have explored the prognosis importance of NMM in patients diagnosed with cancers other than lung cancer. Wang and colleagues conducted a meta-analysis to investigate the prognostic significance of NMM in breast cancer patients who underwent surgical treatment. Pooling data from 29 studies involving 105,600 patients, their study revealed that the presence of NMM in axillary lymph node specimens was associated with poorer five-year disease-free survival (RR = 0.930; 95% CI = 0.907–0.954) and overall survival rates (RR = 0.972; 95% CI = 0.954–0.990) (18). Currently, the staging and treatment guidelines for breast cancer consider the presence of NMM in patients who have undergone axillary lymph node dissection. Moreover, NMM is considered as one of the factors in determining the necessity of adjuvant systemic treatment.

Studies have explored the impact of NMM on oncological outcomes in patients with lung cancer. A study from Japan conducted by Kawano et al. investigated the impact of NMM on oncological outcomes in patients with left-sided lung cancer who underwent initial treatment with surgery. The study included 49 patients, among whom 13 (26.5%) were found to have NMM. The five-year survival rate for patients in this group was 74%, which did not exhibit statistically significant differences compared to patients without NMM (19). Another study from Italy investigated the impact of NMM in stage 1 lung cancer patients undergoing surgical treatment. Examining data from 87 individuals, they found NMM in 14 cases (16%). However, no significant differences were observed in recurrence time, disease-free survival, or overall survival between patients with and without NMM (20). Nevertheless, other studies have found that the presence of NMM affects oncological outcomes, including increased recurrence rates and decreased overall survival, when compared to patients without NMM (12, 13).

In this study, three distinct antigens were employed for the detection of NMM in patients with resectable NSCLC at all stages. It was observed that there were no significant differences in disease-free survival (HR: 0.98, 95% CI: 0.31-3.08, P=0.973) and overall survival (HR: 1.11, 95% CI: 0.23-5.42, P=0.900) between pN0 patients with and without nodal micrometastasis. This is in contrast to Deng et al.’s 2016 meta-analysis, which highlighted NMM in resectable NSCLC as associated with reduced disease-free survival (HR: 2.34, 95% CI: 1.67-3.27, p<0.00001) and 5-year survival rates (HR: 1.98, 95% CI: 1.50-2.62, p<0.00001) (21). It is possible that the sample size in this study is relatively small compared to the sample group from the meta-analysis, and the majority of the meta-analysis sample group is typically in stage 1, resulting in differences in study outcomes. However, when we conducted a subgroup analysis of patients with tumors smaller than 4 cm (stage 1A-1B), the presence of NMM had a negative impact on tumor recurrence (HR: 8.28, 95% CI: 1.64-41.70, P=0.010) and tended to reduce overall survival after surgery (HR: 6.83, 95% CI: 0.76-61.74, P=0.087). The results of this subgroup analysis make the patient group more comparable to the meta-analysis group and yield more consistent study outcomes.

According to current data, the impact of NMM in operable non-small cell lung cancer on cancer-related outcomes remains debatable, encompassing both disease-free survival and overall survival. If NMM does indeed plays a role in patient prognosis, it it might require a reconsideration of tissue examination protocols, emphasizing the use of techniques such as immunohistochemical staining or reverse transcription-polymerase chain reaction (RT-PCR) for more precise nodal staging, consequently guiding more personalized treatment approaches in subsequent management.

At present, studies have been conducted on non-invasive techniques for detecting micrometastases, not only limited to lymph nodes but also including distant metastases. Identifying micrometastases in lymph nodes or other organs prior to treatment can significantly aid in treatment planning. For instance, it can guide lymph node biopsy before surgery or inform decisions regarding peri-operative systemic treatment. Such non-invasive techniques include the use of artificial intelligence (AI) to analyze the characteristics of micrometastases in various tissues from large amounts of imaging data (Radiomics) and the detection of micrometastases through surface-enhanced Raman scattering (SERS). The latter is based on the principle that different mediums exhibit unique scattering patterns when exposed to light energy, allowing for the detection of micrometastases in diverse tissues. Other methods include liquid biopsy and the use of indocyanine green (ICG). However, these methods are still in the research phase and are not yet specifically tailored for detecting nodal micrometastases. Nevertheless, if successfully implemented, they are expected to provide substantial benefits for cancer treatment.

This study may have certain limitations, such as its retrospective nature, which introduces inherent biases. Furthermore, the relatively small sample size may compromise the study’s power to detect differences adequately. Therefore, to provide clearer answers to this issue, prospective multicenters studies with larger sample sizes are necessary.

## Conclusion

Nodal-micro metastasis was identified in 21.43% of pN0 resectable NSCLC patients. Anti-cytokeratin antibody staining is recommended as the primary method for detecting nodal micrometastasis due to its high detection rate. Nonetheless, the benefits of NMM detection in resectable cases remain contentious due to the predominance of retrospective studies, relatively small sample size and conflicting results. Thus, it is recommended to conduct prospective multicenters studies with larger sample sizes to gain a more comprehensive understanding of disease prognosis and its implications for treatment strategy.

## Data Availability

The raw data supporting the conclusions of this article will be made available by the authors, without undue reservation.

## Supplementary material

Supplementary Table 1 Lymph node micro-metastasis by patients.

Supplementary Table 2 Univariable analysis of disease-free survival and overall survival. HR, hazard ratio; CI, confidentail interval; pN0, pathological lymph node negative; LN, lymph node; NMM, nodal micrometastasis; pN1, pathological N1 positive; pN2, pathological N2 positive; CA, carcinoma; diff, differentiation; Ref, reference. **p* values were calculated using the Cox-proportional hazard model.

Supplementary Table 3 Multivariable analysis of disease-free survival and overall survival. HR, hazard ratio; CI, confidentail interval; pN0, pathological lymph node negative; LN, lymph node; NMM, nodal micrometastasis; pN1, pathological N1 positive; pN2, pathological N2 positive; CA, carcinoma; diff, differentiation; Ref, reference. **p* values were calculated using the Cox-proportional hazard model.

Supplementary Table 4 Subgroup analysis of tumor recurrence and overall survival in tumor less than 4 centimeters. pN0, pathological lymph node negative; pN+, pathological lymph node positive; Ref, reference. **p* values were calculated using the Cox-proportional hazard model.
