# Supplementary material for: Prognosis of nodal micrometastasis in resectable pN0 non-small cell lung cancer
Source: Front Oncol. 2025 Jan 31;15:1424682. doi: 10.3389/fonc.2025.1424682 (PMC11825345; doi:10.3389/fonc.2025.1424682)
Supplement: Supplementary Table 1 — Lymph node micro-metastasis by patients. [file Table1.docx]

**Supplement table**

Table 1: lymph node micro-metastasis by patients

| Patient number | LN station | AE1/AE3 | P53 | BerEp4 |
| --- | --- | --- | --- | --- |
| 1 | 8 | + | + |  |
| 2 | 9 | + |  |  |
| 3 | 10 | + | + | + |
| 4 | 7 | + |  |  |
| 5 | 7 | + | + | + |
| 6 | 7 | + | + |  |
| 7 | 7 | + |  |  |
| 8 | 8 | + | + |  |
| 9 | 7 | + | + | + |
| 10 | 7 | + | + |  |
| 11 | 4 | + | + |  |
| 12 | 7 | + | + |  |
| 13 | 7 | + | + |  |
| 14 | 7 | + |  |  |
| 15 | 7 | + |  |  |
| 16 | 7 | + |  | + |
|  | 10 | + |  | + |
| 17 | 7 |  |  | + |
|  | 8 | + | + | + |
| 18 | 7 | + | + | + |
|  | 7 | + | + | + |
| 19 | 7 | + | + | + |
|  | 10 | + |  |  |
| 20 | 10 | + | + |  |
|  | 11 |  |  | + |
| 21 | 8 | + |  |  |
|  | 9 | + |  |  |
| total |  | 25 | 15 | 12 |

Table2: Univariable analysis of disease-free survival and overall survival

| characteristic | Disease-free survival | | | Overall survival | | |
| --- | --- | --- | --- | --- | --- | --- |
|  | HR | 95% CI | P value | HR | 95% CI | P value |
| Age | 1.01 | 0.99-1.02 | 0.531 | 1.01 | 0.98-1.03 | 0.540 |
| Male gender | 1.76 | 1.22-2.55 | 0.002 | 1.86 | 1.16-2.99 | 0.010 |
| Charlson index score | 1.08 | 0.94-1.24 | 0.295 | 1.08 | 0.91-1.28 | 0.365 |
| Tumor size | 1.12 | 1.03-1.22 | 0.009 | 1.11 | 1.00-1.22 | 0.051 |
| Type of resection | 0.96 | 0.68-1.36 | 0.811 | 1.10 | 0.69-1.74 | 0.699 |
| Histology |  |  |  |  |  |  |
| -Adeno CA | Ref. |  |  | Ref. |  |  |
| -Squamous | 1.07 | 0.68-1.69 | 0.759 | 1.14 | 0.64-2.01 | 0.658 |
| -Other CA | 1.34 | 0.54-3.32 | 0.521 | 1.12 | 0.35-3.58 | 0.851 |
| Tumor differentiation |  |  |  |  |  |  |
| -well diff. | Ref |  |  | Ref. |  |  |
| -moderate diff. | 1.17 | 0.76-1.79 | 0.486 | 1.22 | 0.70-2.12 | 0.480 |
| -poorly diff. | 1.34 | 0.79-2.27 | 0.278 | 1.51 | 0.78-2.92 | 0.220 |
| Intra-tumoral vascular invasion | 1.87 | 1.30-2.69 | 0.001 | 1.55 | 0.98-2.45 | 0.062 |
| Intra-tumoral lymphatic invasion | 1.78 | 0.93-3.42 | 0.082 | 1.26 | 0.62-2.54 | 0.521 |
| Pleural invasion | 1.32 | 1.05-1.66 | 0.018 | 1.28 | 0.97-1.69 | 0.085 |
| Perineural invasion | 1.91 | 0.91-4.02 | 0.088 | 1.68 | 0.59-4.75 | 0.328 |
| Presence of tumor necrosis | 2.54 | 1.47-4.37 | 0.001 | 2.78 | 1.37-5.65 | 0.005 |
| LN status |  |  |  |  |  |  |
| -pN0 without NMM | 1.00 | Ref. | Ref. | 1.00 | Ref. | Ref. |
| -pN0 with NMM | 1.66 | 0.78-3.53 | 0.189 | 0.56 | 0.13-2.46 | 0.441 |
| -pN1 | 2.05 | 1.11-3.77 | 0.021 | 3.21 | 1.59-6.48 | 0.001 |
| -pN2 | 2.56 | 1.55-4.22 | <0.001 | 2.70 | 1.46-4.97 | 0.002 |
| Adjuvant treatment | 1.21 | 0.81-1.79 | 0.351 | 1.34 | 0.83-2.18 | 0.200 |

HR, hazard ratio; CI, confidentail interval; pN0, pathological lymph node negative; LN, lymph node; NMM, nodal micrometastasis; pN1, pathological N1 positive; pN2, pathological N2 positive; CA, carcinoma; diff, differentiation; Ref, reference.

**p* values were calculated using the Cox-proportional hazard model

Table3: multivariable analysis of disease-free survival and overall survival

| characteristic | Disease-free survival | | | Overall survival | | |
| --- | --- | --- | --- | --- | --- | --- |
|  | HR | 95% CI | P value | HR | 95% CI | P value |
| Age | 1.01 | 0.96-1.07 | 0.607 | 1.02 | 0.95-1.11 | 0.572 |
| Male gender | 0.81 | 0.36-1.84 | 0.614 | 1.30 | 0.42-4.02 | 0.645 |
| Charlson index score | 1.36 | 0.99-1.87 | 0.058 | 1.35 | 0.95-2.14 | 0.208 |
| Tumor size | 1.28 | 1.04-1.56 | 0.016 | 1.54 | 1.15-2.06 | 0.004 |
| Tumor differentiation |  |  |  |  |  |  |
| -well diff. | Ref |  |  | Ref. |  |  |
| -moderate diff. | 0.43 | 0.17-1.07 | 0.069 | 0.24 | 0.07-0.90 | 0.034 |
| -poorly diff. | 0.91 | 0.18-4.39 | 0.903 | 0.78 | 0.09-6.95 | 0.822 |
| Intra-tumoral vascular invasion | 1.07 | 0.45-2.54 | 0.883 | 1.36 | 0.46-4.03 | 0.582 |
| Intra-tumoral lymphatic invasion | 0.59 | 0.22-1.58 | 0.295 | 0.23 | 0.07-0.78 | 0.018 |
| Pleural invasion | 3.77 | 1.66-8.54 | 0.001 | 4.9 | 1.69-14.20 | 0.003 |
| Perineural invasion | 1.72 | 0.54-5.44 | 0.359 | 3.16 | 0.65-15.25 | 0.152 |
| Presence of tumor necrosis | 2.39 | 0.88-6.51 | 0.087 | 2.40 | 0.61-9.41 | 0.211 |
| LN status |  |  |  |  |  |  |
| -pN0 without NMM | 1.00 | Ref. | Ref. | 1.00 | Ref. | Ref. |
| -pN0 with NMM | 1.68 | 0.74-3.83 | 0.217 | 0.49 | 0.10-2.28 | 0.360 |
| -pN1 | 2.70 | 1.34-5.44 | 0.005 | 4.20 | 1.87-9.45 | 0.001 |
| -pN2 | 3.42 | 1.92-6.10 | <0.001 | 3.31 | 1.65-6.63 | 0.001 |
| Adjuvant treatment | 0.50 | 0.20-1.25 | 0.137 | 0.25 | 0.06-0.97 | 0.045 |

HR, hazard ratio; CI, confidentail interval; pN0, pathological lymph node negative; LN, lymph node; NMM, nodal micrometastasis; pN1, pathological N1 positive; pN2, pathological N2 positive; CA, carcinoma; diff, differentiation; Ref, reference.

**p* values were calculated using the Cox-proportional hazard model

Table 4: Subgroup analysis of tumor recurrence and overall survival in tumor less than 4 centimeters

| Characteristic | Adjusted hazard ratio | 95% confidential interval | P value* |
| --- | --- | --- | --- |
| Tumor recurrence |  |  |  |
| -pN0 without micro-  metastasis    -pN0 with micro-  metastasis    -pN+ | 1.00  8.28    6.58 | Ref.  1.64-41.70  2.97-15.80 | Ref.  0.010  <0.001 |
| Overall survival |  |  |  |
| -pN0 without micro-  metastasis    -pN0 with micro-  metastasis    -pN+ | 1.00  6.83  8.83 | Ref.  0.76-61.74  3.17-24.65 | Ref.  0.087  <0.001 |

pN0, pathological lymph node negative; pN+, pathological lymph node positive; Ref, reference

**p* values were calculated using the Cox-proportional hazard model
